# Impact of Screening Mammography on Breast Cancer Outcomes in Women Aged 80 Years and Over

**DOI:** 10.1245/s10434-025-18288-4

**Published:** 2025-09-13

**Authors:** Siu-Yuan Huang, Makaelah Murray, Angelique Rubio, Nneoma Okoro, Mina S. Sedrak, Susan A. McCloskey, Nicholas Jackson, Mediget Teshome, Nimmi S. Kapoor

**Affiliations:** 1 Department of Surgery, Division of Surgical Oncology, University of California, Los Angeles, Los Angeles, CA USA; 2 David Geffen School of Medicine, University of California, Los Angeles, Los Angeles, CA USA; 3 Division of Health Services Research, David Geffen School of Medicine, University of California, Los Angeles, Los Angeles, CA USA; 4 Department of Medicine, Division of Hematology and Oncology, University of California, Los Angeles, Los Angeles, CA USA; 5 Department of Radiation Oncology, University of California, Los Angeles, Los Angeles, CA USA

**Keywords:** Breast cancer, Screening mammography, Breast cancer screening, Patient outcomes, Advanced age

## Abstract

**Introduction:**

Age remains a significant risk factor for breast cancer, yet specific breast cancer screening guidelines for women > 75 years of age are not clearly defined. We sought to compare differences in outcomes among breast cancer patients diagnosed at ≥ 80 years of age based on receipt of screening mammography.

**Methods:**

This single-institution retrospective review identified breast cancer patients diagnosed at ≥ 80 years of age from 2013 to 2020. The screened cohort underwent screening mammography within 2 years of diagnosis. Characteristics of the screened/unscreened cohorts were compared using Chi-square and *t*-tests. Kaplan–Meier survival analysis and log-rank testing were performed to compare overall survival (OS) and disease-free survival (DFS). Cox proportional hazard models produced unadjusted/adjusted hazard ratios (HRs) to estimate the association of receiving a screening mammogram with OS/DFS.

**Results:**

Of 174 patients, 98 were screened and 76 were unscreened. Median age was 83 years, most patients had stage I/II tumors, and most cancers were estrogen receptor-positive/human epidermal growth factor receptor 2-negative. The groups did not significantly differ in race/ethnicity, comorbidities, receptor subtype, axillary surgery, or receipt of endocrine therapy/chemotherapy. Unscreened patients were more likely to have tumors that were palpable, high grade, and advanced stage. More screened patients underwent lumpectomy, while more unscreened patients omitted surgery. With a median follow-up of 55 months, the screened cohort had improved DFS (HR 0.45, 95% confidence interval [CI] 0.301–0.665; *p* < 0.001) and OS (HR 0.26, 95% CI 0.126–0.544; *p* < 0.001). This persisted when adjusted for age, receptor subtype, and surgery.

**Conclusions:**

Breast cancer patients diagnosed at ≥ 80 years of age who received screening mammography presented with earlier-stage disease and had improved DFS and OS compared with the unscreened cohort.

Between 2010 and 2020, the population of adults aged 65 years and older in the United States (US) experienced the fastest growth rate in over a century.^1^ This older population increased by 15.5 million people and became 16.8% of the total population. Older age is a known risk factor for breast cancer, and as the aging US population continues to grow, uncertainties about screening mammography in the elderly will need to be addressed. There is a lack of consensus among multiple organizations regarding breast cancer screening guidelines in the elderly. Currently, the United States Preventive Services Task Force (USPSTF) does not have formal recommendations for screening mammograms in women aged 75 years or older, citing insufficient evidence.^2^ The American Cancer Society recommends that breast cancer screening should continue if a patient is in good health and has a life expectancy of 10 years or more. Similarly, the American College of Radiology recommends that screening continues as long as women are willing to undergo screening and any subsequent biopsies, and if their overall health remains good.^3^

However, with increasing age, elderly patients may have more comorbidities that could pose more of a threat to their overall health than breast cancer. This raises the question of whether screening mammography is beneficial in elderly patients with multiple comorbidities. Studies of elderly breast cancer patients have led to de-escalation of care, most notably the Choosing Wisely campaign, wherein the Society of Surgical Oncology stated in 2016, “Don’t routinely use sentinel lymph node biopsy in clinically node-negative women 70 years of age with hormone receptor-positive invasive breast cancer.”^4^ While there is strong evidence for treating breast cancer less aggressively in the elderly community without impact on survival, it remains to be seen if screening guidelines can also be relaxed in this patient population.

Given the limited data regarding the benefit of screening mammography in elderly women, and if or when they can safely cease breast cancer screening, we sought to identify differences in outcomes between screened and unscreened women diagnosed with breast cancer at age 80 years or older.

## Methods

Patients diagnosed with breast cancer at age 80 years and older at the University of California, Los Angeles (UCLA) between 2013 and 2020 were sourced from the UCLA Health electronic health record system for this retrospective cohort study. Patients with a prior breast cancer diagnosis or who were missing significant data that would preclude analysis (such as age at diagnosis or date of core needle biopsy) were excluded. The screened cohort was defined as patients who received a screening mammogram within 2 years of diagnosis.

Demographic variables included current age, sex, race, ethnicity, smoking status, marital status, primary language, Area Deprivation Index (ADI) at both the state and national levels, the total number of documented health issues in their problem list, which includes past medical history within 3 months of core needle biopsy, and the presence of pre-existing hypertension and/or diabetes at the time of breast cancer diagnosis determined by the date of core biopsy. ADI rankings were obtained by entering patient addresses into the Neighborhood Atlas Tool (https://www.neighborhoodatlas.medicine.wisc.edu/). The number of health problems was recorded from the problem list within 3 months of the core biopsy date.

Data on cancer diagnosis included age at diagnosis (determined by the date of core biopsy), method of detection, and symptoms, including palpable findings, nipple discharge, breast pain, and breast skin changes.

Treatment data included whether patients underwent surgery and if so, the type of breast and nodal surgery. Neoadjuvant and adjuvant treatments were documented. The last follow-up date was defined as the patient's most recent office visit with any provider. Recurrence data included the type (locoregional or metastatic), date, date of death (if applicable), and whether the cause of death was attributed to breast cancer.

Tumor characteristics were extracted from surgical pathology reports, or core biopsy reports if surgery was not performed. For patients with more than one tumor type, data on the largest invasive tumor were prioritized.

Characteristics of the screened cohort were compared with the unscreened cohort using Chi-square and *t*-tests. Kaplan–Meier survival analysis and log-rank testing was performed to compare overall survival (OS) and disease-free survival (DFS). Hazard ratios (HRs) for patients who received a screening mammography were estimated using Cox proportional hazards models, including unadjusted and adjusted for covariates. We included the following covariates in the Cox regression model: age at diagnosis (continuous), invasive tumor subtype (estrogen receptor [ER]+/human epidermal growth factor receptor 2 [HER2]−, ER−/HER2+, ER+/HER2+, triple-negative breast cancer [TNBC]), and surgery as treatment (yes/no). Covariates were selected based on their established clinical relevance.

This study was found exempt by the UCLA Health Institutional Review Board and a request to waive informed consent was granted. Statistical analyses were conducted in R version 4.4.1 (http://www.r-project.org).

## Results

### Patient Characteristics

**Fig. 1:**
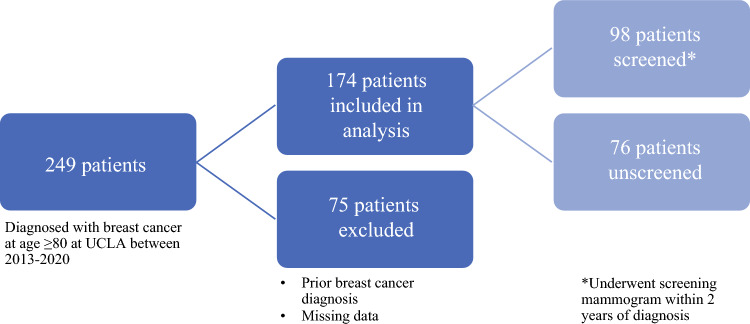
Patient population, with inclusion/exclusion criteria. *UCLA* University of California, Los Angeles

A total of 249 patients were initially identified. Of the 174 patients included in analysis, 98 (56.3%) were in the screened cohort and 76 (42.7%) were unscreened (Fig. 1). The median patient age was 83 years (range 80–98). A majority of the patients were White (70%) and not Hispanic or Latino (89%). The two groups did not differ significantly in race, ethnicity, marital status, smoking status, ADI, rates of hypertension and diabetes mellitus, or number of comorbidities (as represented by the number of items on their problem list). The screened group had a lower median age at diagnosis than the unscreened group (83 years vs. 85 years; *p* = 0.001). The unscreened group had a higher rate of symptomatic tumors (88% vs. 34%; *p* < 0.001), including tumors detected by palpation (79% vs. 25%; *p* < 0.001). Patient characteristics of the two groups are listed in Table 1.

**Table 1:** Patient characteristics

Characteristic	Overall patients [*n* = 174]	No screening mammogram [*n* = 76]	Screening mammogram [*n* = 98]	*p* Value
Age at diagnosis, years				0.024
80–84	104 (60)	37 (36)	67 (64)	
85–89	51 (29)	27 (53)	24 (47)	
90+	19 (11)	12 (63)	7 (37)	
Median (IQR)	83.0 (81.0, 87.0)	85.0 (82.0, 89.0)	83.0 (81.0, 86.0)	0.001
Sex				0.437
Female	173 (99)	75 (43)	98 (57)	
Male	1 (0.6)	1 (100)	0 (0)	
Race				0.655
Native American	2 (1.1)	1 (50)	1 (50)	
Asian	11 (6.3)	4 (36)	7 (64)	
Black	17 (9.8)	5 (29)	12 (71)	
Other	16 (9.2)	9 (56)	7 (44)	
Unknown	7 (4.0)	4 (57)	3 (43)	
White	121 (70)	53 (44)	68 (56)	
Ethnicity				0.661
Hispanic or Latino	10 (5.7)	5 (50)	5 (50)	
Non-Hispanic or Latino	155 (89)	66 (43)	89 (57)	
Unknown	9 (5.2)	5 (56)	4 (44)	
Smoking status				0.728
Yes	7 (4.0)	4 (57)	3 (43)	
Former	24 (14)	10 (42)	14 (58)	
No	140 (80)	60 (43)	80 (57)	
Unknown	3 (1.7)	3 (1.7)	2 (67)	
Marital status				0.409
Divorced	14 (8.0)	5 (36)	9 (64)	
Married	45 (26)	17 (38)	28 (62)	
Single	27 (16)	14 (52)	13 (48)	
Unknown	2 (1.1)	2 (100)	0 (0)	
Widowed	86 (49)	38 (44)	48 (56)	
ADI (median, IQR)	4 (1, 11)	5 (2, 12)	3 (1, 10)	0.079
Symptomatic				<0.001
Yes	100 (57)	67 (67)	33 (33)	
No	69 (40)	7 (10)	62 (90)	
Unknown	5 (2.9)	2 (40)	3 (60)	
Detected by palpation				<0.001
Yes	85 (49)	60 (71)	25 (29)	
No	83 (48)	13 (16)	70 (84)	
Unknown	4 (2.3)	2 (50)	2 (50)	
Problem list length (median, IQR)	9 (3, 19)	9 (4, 19)	9 (3, 19)	0.985
Hypertension				0.777
Yes	124 (71)	55 (44)	69 (56)	
No	50 (29)	21 (42)	29 (58)	
Diabetes				0.257
Yes	26 (15)	14 (54)	12 (46)	
No	148 (85)	62 (42)	86 (58)	

Data are expressed as *n* (%) unless otherwise specified

*ADI* Area Deprivation Index, *IQR* interquartile range

### Tumor Characteristics

Among the 174 patients, there were 181 total cancer diagnoses, with 7 patients having bilateral cancers. The majority of tumors were invasive ductal carcinoma (64%) and the median tumor size was 2.1 cm. The majority (121/156, 78%) of the cohort with invasive tumors presented with ER+ and HER2− tumors. 19/156 (12%) patients with invasive tumors had HER2+ disease, and 16/156 (10%) had triple-negative disease. The unscreened group was more likely to have invasive ductal carcinoma, while the screened group was more likely to have ductal carcinoma in situ (DCIS) [*p* = 0.037]. The unscreened group also presented with larger tumors (median 3.1 cm vs. 1.7 cm; *p* < 0.001), higher-grade tumors (*p* < 0.001), and more advanced stage (*p* < 0.001). The tumors in the screened and unscreened groups did not differ in ER or progesterone receptor (PR) positivity, HER2 status, or invasive tumor subtype. Tumor and treatment characteristics of the two groups are listed in Table 2.

**Table 2:** Tumor and treatment characteristics

Characteristic	Overall observations (*n* = 181)	No screening mammogram (*n* = 80)	Screening mammogram (*n* = 101)	*p*-Value
Histology				0.037
DCIS	25 (14)	7 (28)	18 (72)	
IDC	116 (64)	60 (52)	56 (48)	
ILC	26 (14)	7 (27)	19 (73)	
Other	14 (7.7)	6 (43)	8 (57)	
Size, cm				
Median (IQR)	2.10 (1.10, 3.70)	3.10 (1.50, 4.30)	1.70 (0.90, 2.90)	<0.001
Grade [*n* = 163]				<0.001
1	50 (30)	14 (28)	36 (72)	
2	71 (43)	30 (42)	41 (58)	
3	42 (26)	28 (67)	14 (33)	
Stage [*n* = 177]				<0.001
0	24 (13)	7 (29)	17 (71)	
1	70 (39)	19 (27)	51 (73)	
2	59 (33)	33 (56)	26 (44)	
3	24 (13)	17 (71)	7 (29)	
4	4 (2.2)	4 (100)	0 (0)	
ER status [*n* = 175]				0.510
Negative	25 (14)	13 (52)	12 (48)	
Positive	149 (85)	63 (42)	86 (58)	
Unknown	1 (0.6)	0 (0)	1 (100)	
PR status [*n* = 175]				0.586
Negative	43 (25)	21 (49)	22 (51)	
Positive	131 (75)	55 (42)	76 (58)	
Unknown	1 (0.6)	0 (0)	1 (100)	
HER2 IHC [*n* = 156]				0.530
Equivocal	35 (22)	19 (54)	16 (46)	
Negative	110 (71)	46 (42)	64 (58)	
Positive	9 (5.8)	5 (56)	4 (44)	
Unknown	2 (1.3)	1 (50)	1 (50)	
HER2 FISH [*n* = 141]				0.497
Equivocal	1 (0.7)	0 (0)	1 (100)	
Negative	120 (85)	52 (43)	68 (57)	
Positive	17 (12)	10 (59)	7 (41)	
Unknown	3 (2.1)	1 (33)	2 (67)	
Invasive tumor subtype [*n* = 156]				0.406
ER+/HER2−	121 (78)	53 (44)	68 (56)	
ER−/HER2+	4 (2.6)	2 (50)	2 (50)	
ER+/HER2+	15 (9.6)	10 (67)	5 (33)	
Triple negative	16 (10)	7 (44)	9 (56)	
Nodal surgery				0.167
ALND	2 (1.1)	1 (50)	1 (50)	
SLNB	83 (46)	30 (36)	53 (64)	
SLNB AND ALND	5 (2.8)	2 (40)	3 (60)	
None	91 (50)	47 (50)	44 (48)	
Type of surgery				<0.001
Breast-conserving surgery	128 (71)	43 (34)	85 (66)	
Mastectomy	34 (19)	23 (68)	11 (32)	
None	19 (10)	14 (74)	5 (26)	
Reconstruction [*n* = 158]				0.402
Yes	6 (3.8)	1 (17)	5 (83)	
No	152 (96)	63 (41)	89 (59)	

Characteristic	Overall patients (*n* = 174)	No screening mammogram (*n* = 76)	Screening mammogram (*n* = 98)	*p*-Value
Neoadjuvant chemotherapy				0.003
Yes	23 (13)	17 (74)	6 (26)	
No	151 (87)	61 (40)	90 (60)	
Adjuvant chemotherapy				0.638
Yes	4 (2.3)	1 (25)	3 (75)	
No	170 (98)	73 (43)	97 (57)	
Radiation				0.003
Yes	59 (34)	16 (27)	43 (73)	
No	115 (66)	58 (50)	57 (50)	
Trastuzumab				0.150
Yes	13 (7.5)	8 (62)	5 (38)	
No	161 (93)	66 (41)	95 (59)	

Data are expressed as *n* (%) unless otherwise specified

*DCIS* ductal carcinoma in situ, *IDC* invasive ductal carcinoma, *ILC* invasive lobular carcinoma, *ER* estrogen receptor, *PR* progesterone receptor, *HER2* human epidermal growth factor receptor, *IHC* immunohistochemistry, *FISH* fluorescence in situ hybridization, *ALND* axillary lymph node dissection, *SLNB* sentinel lymph node biopsy, *IQR* interquartile range

### Treatment Characteristics

Overall, 71% of patients were treated with breast-conserving surgery, although notably 10% were treated without surgery. Nodal surgery was not performed for 50% of patients. The majority of patients did not undergo neoadjuvant or adjuvant chemotherapy (87% and 98%, respectively). Most patients (115/174, 66%) did not undergo radiation therapy. The screened patients were more likely to undergo breast-conserving surgery (82.4% vs. 60.6%; *p* < 0.001) and radiation (43% vs. 20%; *p* = 0.003), while unscreened patients were more likely to undergo mastectomy or no surgery at all (38.8% vs. 17.6%; *p* = 0.009). While only 13% of patients overall received neoadjuvant chemotherapy, the unscreened group made up a higher proportion of these patients (74% were unscreened, 26% were screened; *p* = 0.003). The two groups did not differ in terms of type of nodal surgery, rates of reconstruction, treatment with adjuvant chemotherapy, or treatment with trastuzumab. Of the 121 ER+/HER2− patients, 70 (58%) took endocrine therapy, 49 (40%) did not take endocrine therapy, and the remaining 2 (1.7%) patients were unknown. Regarding nodal surgery in the same population, 58 (48%) patients had sentinel lymph node biopsy (SLNB) only, 4 (3%) had SLNB and axillary lymph node dissection (ALND), and 59 (49%) did not have any nodal surgery.

### Survival Outcomes

**Fig. 2:**
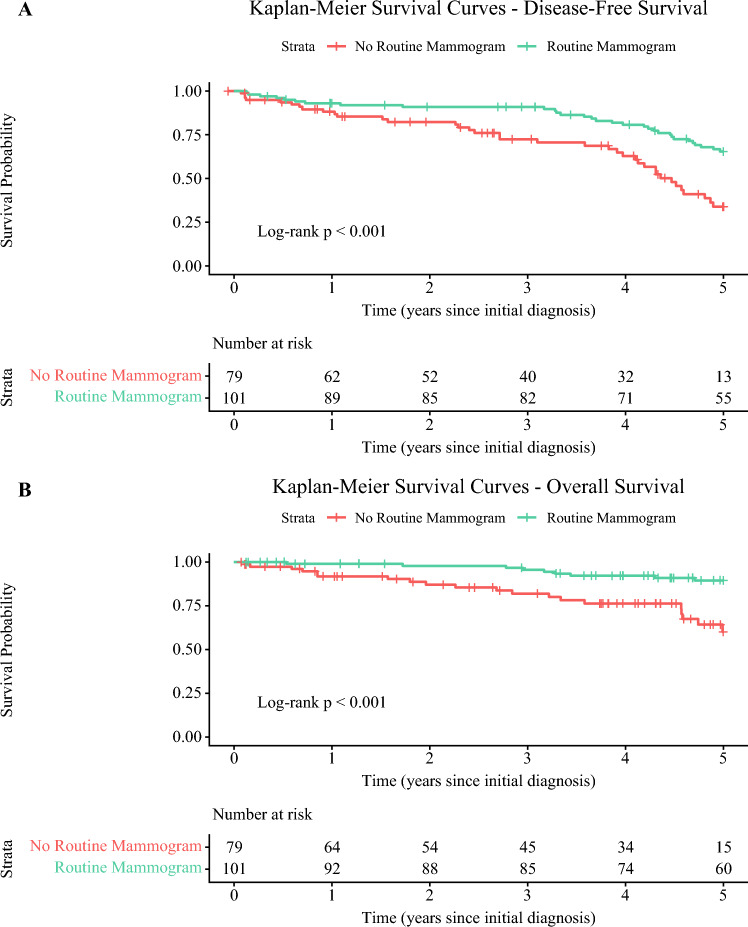
**a** Kaplan–Meier survival curves comparing disease-free survival between patients with and without routine mammogram screening. The curves are truncated at 5 years because the number of patients at risk in the non-routine screening group fell below 10 beyond this time point, reducing the reliability of survival estimates. The risk table shows the number of patients at risk at each 1-year interval. The log-rank test indicates a significant difference between groups (*p* < 0.001). **b** Kaplan–Meier survival curves comparing overall survival between patients with and without routine mammogram screening. The curves are truncated at 5 years because the number of patients at risk in the non-routine screening group fell below 10 beyond this time point, reducing the reliability of survival estimates. The risk table shows the number of patients at risk at each 1-year interval. The log-rank test indicates a significant difference between groups (*p* < 0.001)

Median follow-up for the cohort was 55 months (range 1–146 months), with 29 recurrences (16.7%) and 36 deaths (20.7%) recorded. Compared with the unscreened group, the screened group had improved DFS (HR 0.439, 95% confidence interval [CI] 0.296–0.653; *p* < 0.001) and OS (HR 0.275, 95% CI 0.135–0.561; *p* < 0.001) (Fig. 2). When adjusted for age, invasive tumor subtype, and surgery, the benefits in DFS (HR 0.376, 95% CI 0.236–0.598; *p* < 0.001) and OS (HR 0.417, 95% CI 0.181–0.959; *p* = 0.039) persisted in the screened cohort.

## Discussion

In this study of 174 elderly women diagnosed with breast cancer at age 80 years or older, we compared outcomes between women who had screening mammography within 2 years of diagnosis with those who did not undergo screening. The unscreened group was more likely to present with larger tumors that were higher grade and at a more advanced stage. The screened group was found to have improved DFS and OS compared with the unscreened group, which persisted after adjustment for age, invasive tumor subtype, and receipt of surgery.

Our results are similar to that of a 2014 study that included 1914 women participating in the Women’s Health Initiative and diagnosed with breast cancer at age 75 years or older, stratified by time since their last mammogram prior to diagnosis.^5^ Compared with those who had a mammogram within 2 years of diagnosis, those who had a mammogram between 2 and 5 years or more than 5 years prior to diagnosis had an increased risk of breast cancer mortality. Interestingly, there was no difference in all-cause mortality between the three groups.^5^ A similar study looking at breast cancer patients from New Zealand with a lower age cut-off of 70 years also noted improved disease-specific and OS in those with screen-detected cancers.^6^ A 2017 National Mammography Database study noted that screening performance of mammography improves with age, specifically a lower recall rate, increased cancer detection rate, and increased positive predictive values for biopsy recommendation and biopsy performance.^7^ They cited decreasing breast density with age as a contributor, but other factors may include fewer hormonal factors and greater availability of prior studies for comparison in this age group. A study using the Vermont Breast Cancer Surveillance System had similar findings, with increasing sensitivity and positive predictive value with increasing patient age, stratified by decade.^8^ If a patient can tolerate mammography, there does not appear to be strong evidence for a definite age cut-off to cease breast cancer screening; however, the influence of primary care providers and a commitment to shared decision making are important factors in the patient’s ultimate decision.^9,10^ While there is no consensus among national guidelines whether patients over 75 years of age should continue screening mammograms, there is even less data on what the frequency of mammograms should be in this population. Zuley et al. found that women who underwent annual screening had a significantly lower percentage of late-stage breast cancers and improved OS compared with those who had biennial or intermittent screening, but only 6% of their cohort was aged 80 years or older.^11^

Currently, annual mammography is generally recommended to continue while patients remain in good health. Our screened and unscreened cohorts were relatively well-matched in terms of comorbidities. It is likely that the decision for an elderly patient to receive screening mammograms is multifactorial, including, but not limited to, patient preference, primary care doctor assessment, socioeconomic factors, and patient medical and family history. A study from 2007 surveying patients aged 65 years and older regarding their decision to undergo screening mammogram found that regardless of their choice, the most important factor they considered was their doctor’s recommendation.^10^ Moreover, a specific comorbidity may also cause a provider to advocate for continuing or omitting screening. For example, patients suffering from dementia have been found to undergo lower rates of breast and prostate cancer screening compared with those of a similar age without an impaired cognitive status.^12^

Some concerns regarding continuing breast cancer screening in the elderly include overdiagnosis (diagnosing a cancer that would not have caused symptoms in the patient’s lifetime) and cost effectiveness. A retrospective study of the Surveillance, Epidemiology, and End Results (SEER) database of women aged 70 years and older estimated a 31–54% overdiagnosis rate depending on their specific age range, and did not find a significant decrease in breast cancer-specific death with screening.^13^ A separate study performed a simulation of cost effectiveness for screening mammography in patients aged 75–90 years, stratified by comorbidities. They found that while annual screening was not cost effective, biennial screening until age 80 years was cost effective, although the number of deaths prevented was low.^14^

Although studies have varied in whether elderly patients present with more favorable tumor biology compared with younger patients, the most common type of breast cancer in both age groups is hormone receptor-positive (HR+), HER2− subtype.^15^ Additionally, elderly women are more likely to present with a palpable mass and less likely to be offered screening mammography.^16,17^ Survival from breast cancer is also impacted by older age, and a meta-analysis of 63 studies on breast cancer in elderly patients found that when compared with patients aged 70–79 years, patients aged ≥80 years had higher breast cancer-specific mortality at 5 years (25.8% vs. 17.2%; *p* < 0.01) and 10 years (32.7% vs. 26.6%; *p* < 0.01).^18^ All patients diagnosed with stage IV disease in our cohort were in the unscreened group but made up only 2.3% of the cohort overall (4 patients). Given these small numbers, we are unable to draw conclusions regarding differences in outcomes in specific subgroups such as DCIS (which accounted for 14% of all cancer diagnoses in our study) or stage IV breast cancer patients.

Management of breast cancer in the elderly is a growing field of research as the senior population continues to increase. Meanwhile, the trend in breast cancer treatment in the elderly has been moving towards de-escalation, with omission of SLNB recommended in patients over 70 years of age with a clinically negative axilla and small tumors, with favorable biology per the Choosing Wisely guidelines.^4^ Similarly, the CALGB 9343 study investigated the benefit of post-lumpectomy radiation in women aged 70 years or older with T1N0M0 hormone-positive breast cancers and found a only a small improvement in locoregional recurrence rates with radiation, but no difference in OS, distant DFS, or mastectomy rates after 10 years.^19^ This study led to the National Comprehensive Cancer Network (NCCN) guidelines suggesting omission of radiation in these patients if adjuvant endocrine therapy is planned.

Other areas of treatment de-escalation that are being studied include cryoablation and active surveillance. Notably, the ICE3 trial recently published their 5-year follow-up of 194 women aged 60 years or older with low grade, 1.5 cm or smaller, HR+ and HER2− invasive ductal carcinoma who underwent cryoablation, where they noted a 4.3% recurrence rate and 96.7% breast cancer survival rate.^20^ Active surveillance of DCIS is currently being studied in comparison with guideline-concordant care (surgery with or without radiation) in the COMET trial. Newly published data from the COMET trial showed that in patients aged 40 years or older diagnosed with HR+ grade 1 or 2 DCIS, the active surveillance group did not have a higher rate of Ipsilateral invasive cancer diagnoses compared with the guideline-concordant care group after 2 years of follow-up.^21^ Forty-two percent of patients in the COMET trial were over the age of 65 years, but further information regarding the age distribution of this subgroup is not yet available. In our study, DCIS made up 14% of all cancer diagnoses, the majority of which were in the screened group (72% vs. 28% in the unscreened group). This is intuitive as DCIS generally presents as calcifications rather than a palpable mass.

Lastly, although our screened and unscreened cohorts did not differ significantly in race or ethnicity, racial disparities in breast cancer outcomes have frequently been identified, which may be due in part to disparities in screening. A survey of 600 elderly Hispanic women in Los Angeles found that 74% had never had a mammogram.^22^ A meta-analysis of data spanning from 1946 to 2015 found that Black and Hispanic patients were significantly less likely to utilize screening mammograms compared with White and Asian patients;^23^ however, more recent data show that screening mammogram uptake among Black and Hispanic women in the US is on par with or exceeds that of White women.^24,25^ Despite this, among octogenarians diagnosed with breast cancer, a study utilizing the National Cancer Database (NCDB) found that Black race was an independent risk factor for late-stage at diagnosis, and Black patients were also more likely to present with TNBC.^26^ The racial disparities in breast cancer outcomes are likely multifactorial regardless of age or screening status, and require further study.

Limitations of this study include its retrospective nature and relatively small sample size. While this study took place in a relatively diverse urban setting, the majority of patients were White and non-Hispanic, which may limit its applicability to the greater population. Additionally, this study only included patients diagnosed with breast cancer, and therefore does not address potential downsides of screening mammography in patients not diagnosed with breast cancer. A study of 2011 patients aged 80 years or older found an 11% false positive rate after screening mammogram (meaning additional tests or procedures were done, but did not lead to a diagnosis of breast cancer).^27^ The financial and emotional stress of nondiagnostic biopsies may not only apply to the patients themselves but also their family members or caretakers who may be assisting with transportation and medical care.

## Conclusions

Among our cohort of women aged 80 years or older who were diagnosed with breast cancer, those who received screening mammography within 2 years of diagnosis presented with earlier-stage disease and had improved DFS and OS compared with those who did not. Our study does not support any particular age cut-off for stopping screening mammography. In the context of an aging population, prospective studies to define breast cancer screening in older women are needed to identify which subsets may safely omit screening.
